# Meningo-Cerebritis

**Published:** 1857-09

**Authors:** Benj. Heeton Lemon

**Affiliations:** Buffalo


### BUFFALO MEDICAL JOURNAL

AND

### MONTHLY REVIEW.

**VOL. 13.** SEPTEMBER, 1857. **NO. 4.**

### ORIGINAL COMMUNICATIONS.

ART. I.—*Meningo-Cerebritis. Death, Necropsy, and Microscopic Inspection of Brain. Subject, a girl æt.* 6 *years, nervous temperament, hair and complexion fair, blue eyes, moderate cerebral development, precocious.* Practice of Dr. WILCOX. Reported by BENJ. HEETON LEMON, M. D., Buffalo.

June 30th. Saw Carrie M——, Clinton street. The mother says her child has lately lost her appetite and been feverish and costive Yesterday forenoon began vomiting; had high fever; headache; thirst. To-day has vomiting, stomach rejects everything; tongue furred; bowels constipated; rapid and quick pulse; headache; thirst; general exaltation.

July 1st. Headache, vomiting, &c., had ceased, and she rested comparatively well last night; learned that bleeding at the nose occurred the day previous to the first visit; there is now a sero-sangumolent exudation from, and she is inclined to pick at, and thrust her fingers into it; eyes have a peculiar glistening appearance, pupils natural; no fever, in slight perspiration.

July 2d. Yesterday evening the headache and fever returned with intense severity; she refers the pain to the forehead. This morning there is no pain in the head, and but little heat of surface; head is giddy; speaks quickly; seems inclined to get out of bed; thoughts confused; fretful most of the time, but will laugh or cry without any apparent reason; sighs frequently. Exacerbation of the symptoms occurs periodically every evening, the paroxysms lasting about six hours.

July 3d. Dr. Rochester in consultation.

The symptoms recurred yesterday evening at the usual time, but with less severity than previously; fever and headache remain. Strabismus first noticed by the mother during the night; this morning it is still present in slight degree; head is giddy; singing in the ears; labored and sighing respiration.

4th. Slight improvement; squinting and the other symptoms present, though in less degree than yesterday. The periodical exacerbation occurred again last evening of the usual duration, but no fever, the bowels having been thoroughly evacuated.

Called at 8, P. M., but did not arrive until 3 o'clock in the morning. The noise and excitement of the anniversary had aggravated the symptoms; she had been delirious during the night, but is now in a partially comatose state; would only collect her faculties and answer after considerable time; could slowly protrude her tongue after some length of time and with great apparent effort; when it was out would forget to withdraw it unless reminded, and then very slowly; increased squinting.

5th. Deeper coma; pupils dilated; partial loss of vision; grinding of the teeth; oscillation of eyeballs; muttering.

7, P. M. Coma more profound; entire loss of vision; cannot be roused; cannot put out her tongue; deglutition difficult, whatever is given having to be forced to the posterior pharynx.

6th. In about the same state, with the addition of retention of urine; this was relieved, but paralysis of the bladder succeeded, and the urine subsequently dribbled away involuntarily.

7th. Profound coma; stertorous respiration; convulsions; collapse, and death on the evening of the 8th. Duration of the illness being about ten days.

*Autops. Cadav.*, about forty hours after death. Present, Doctors White, Flint, Rochester, Wilcox, Austin Flint, Jr., and Mr. Harvey.

Incision through integument over vertex to each ear; reflected flaps; applied saw, cutting through outer table and diploe; cracked inner table with chisel and hammer. (By this method the inconveniences of either saw and hammer, and chisel singly, is avoided, there being no danger of injuring the membranes or brain, with greater facility of retaining the calvarium in its place when reäpplied.)

The skull cap being removed the dura mater was found healthy, not more adherent to the bones than natural to the age. Divided at the sides, drawn to the top of brain, brought into view subjacent membranes.

The sac of arachnoid was moist but contained no fluid. Cerebro-spinal fluid in considerable quantity lying under it, in the sulci of convolutions; pia mater highly vascular, and the tissues much engorged with blood. The anterior attachments of the membranes being divided they were drawn backward altogether. The pia mater and arachnoid adhered in a small patch at the anterior superior aspect of the anterior lobe of the right hemisphere, and at the posterior part of posterior lobe of the left hemisphere, there was a patch of quite firm adhesion as large as a quarter of a dollar.

The anterior lobes were then carefully raised, and the several pairs of nerves cut as they were brought into view, finally, the spinal cord, as far down as possible, all of the medulla oblongata being brought away. The brain was then laid on a plate, base upward, those portions of the membranes covering the base were not brought away with it.

The left ventricle was much distended with fluid. The right had none. The quantity of fluid, including that of the ventricle and the cerebro-spinal, was, at a rough estimate, about 2½ ounces.

The region from the optic commissure posteriorly to the pous varolii, and, indeed, all the surface known as the "locus quadri-lateralis, was covered over with exudations of plastic material, the arachnoid corresponding with this surface was thickened and opaque.

The 5th, 3d, and 4th ventricles were not examined with reference to contained fluid. The whole brain was softened, and easily removed with handle of scalpel. No peculiar appearances were observed either in the cineritious or white substances.

The whole was softened equally alike, as tested by Dr. Flint, by slicing and pouring on water.

The examination of the brain and membranes being concluded, the thorax was opened and the lungs, heart, and spleen inspected. Nothing noteworthy observed, but a portion of the apex of the left lung and a piece of the spleen were saved and added to the specimens already preserved from the base of the brain, (these being from the regions already described as the situations of the exudations and the opaque arachnoid) for microscopic examination, by Dr. Austin Flint, Jr., the results of which the following is his written account:

"Examined a portion of the brain taken from the base just back of commissure of the optic nerves, including part of the commissure, about forty hours after death.

"The object of the examination was to determine the presence or absence of tubercle.

"The parts removed were covered with a layer of coagulating lymph of a straw color, translucent, and of the consistence of thick jelly.

"On submitting portions of this and the subjacent tissues, to microscopic examination, in presence of Professors White and Flint, the characteristic anatomical elements of the brain were observed, together wich masses of exudation matter and fatty granules, occasionally inclosing a blood-vessel.

"The so-called inflammatory and exudation corpuscles of Gluge and Bennett were observed scattered over the field.

"The microscopic appearances were characteristic cf inflammation, attended with exudation of lymph, but not dependent upon the presence or deposition of tubercle.

"Repeated and careful examinations were made with reference to the presence of the characteristic tubercular corpuscle, but it was not discovered."

*Recapitulation of Symptoms and order of their Development.* Cephalalgia; accelerated pulse; dry, hot skin; thirst; anorexia; general exaltation of the intellectual faculties; vomiting; furred tongue; constipation; epistaxis; speech quick; incoherent; fretful, will laugh or cry without any apparent cause; sighs frequently; is inclined to get out of bed; a peculiar shining appearance of eyes; strabismus; tinnitus aurium; labored respiration; sighing deeply at short intervals; stupor; delirium; partial coma; loss of memory; confusion of thought; difficult articulation; increased squinting; deeper coma; pupils dilated; partial loss of vision; grinding of the teeth; oscillation of the eyeballs; coma more profound: entire loss of vision; cannot protrude tongue; deglutition difficult; retention of urine; profound coma; stertorous respiration; involuntary discharge of urine; convulsions; collapse.

*Causes.* Over exertion of the mental faculties at school, probably, operated as a predisposing cause in this case.

The early supervention of coma and the prominence of this symptom, together with vomiting, is worthy of note, as particularly diagnostic of the seat and character of the lesion.

"The average duration of meningitis, is according to Solly, about fifteen days. He divides it into three stages:

"The first, manifested by an exaltation of sensibility, hence cephalalgia; if seated at the base of the brain, drowsiness, nausea, vomiting.

"The second stage, the period of reäction, disturbance of muscular system, convulsions, delirium, agitation, contractions, oscillation, dilatation of pupils.

"The third, the shortest and the period of collapse, abolition of the senses, local and general paralysis, coma." — *Solly on the Human Brain.*

